# FLIM FRET Visualization of Cdc42 Activation by Netrin-1 in Embryonic Spinal Commissural Neuron Growth Cones

**DOI:** 10.1371/journal.pone.0159405

**Published:** 2016-08-02

**Authors:** Benjamin Rappaz, Karen Lai Wing Sun, James P. Correia, Paul W. Wiseman, Timothy E. Kennedy

**Affiliations:** 1 Program in NeuroEngineering, McGill University, Montreal, QC, H3A 2B4, Canada; 2 Department of Neurology and Neurosurgery, Montréal Neurological Institute, McGill University, Montreal, QC, H3A 2B4, Canada; 3 Department of Physics, McGill University, Montreal, QC, H3A 2T8, Canada; 4 Department of Chemistry, McGill University, Montreal, QC, H3A 0B8, Canada; School of Biomedical Sciences, The University of Queensland, AUSTRALIA

## Abstract

Netrin-1 is an essential extracellular chemoattractant that signals through its receptor DCC to guide commissural axon extension in the embryonic spinal cord. DCC directs the organization of F-actin in growth cones by activating an intracellular protein complex that includes the Rho GTPase Cdc42, a critical regulator of cell polarity and directional migration. To address the spatial distribution of signaling events downstream of netrin-1, we expressed the FRET biosensor Raichu-Cdc42 in cultured embryonic rat spinal commissural neurons. Using FLIM-FRET imaging we detected rapid activation of Cdc42 in neuronal growth cones following application of netrin-1. Investigating the signaling mechanisms that control Cdc42 activation by netrin-1, we demonstrate that netrin-1 rapidly enriches DCC at the leading edge of commissural neuron growth cones and that netrin-1 induced activation of Cdc42 in the growth cone is blocked by inhibiting src family kinase signaling. These findings reveal the activation of Cdc42 in embryonic spinal commissural axon growth cones and support the conclusion that src family kinase activation downstream of DCC is required for Cdc42 activation by netrin-1.

## Introduction

Netrin-1 protein secreted by floor plate cells functions as a chemoattractant that directs commissural axon extension to the ventral midline of the embryonic vertebrate neural tube [1–5]. While netrin-1 is essential for normal neural development, and many downstream components of netrin-1 signaling have been identified, how the activation of these proteins directs growth cone motility remains incompletely understood.

Cdc42, a member of the Rho family of small GTPases, exhibits a highly conserved capacity to co-ordinate the intracellular signaling mechanisms that underlie directional migration [6]. Biochemical studies of spinal commissural neurons revealed that netrin-1 signals through the transmembrane receptor Deleted in colorectal cancer (DCC) to activate Cdc42 in embryonic rat spinal commissural neurons [7]. Key downstream effectors of Cdc42 include other Rho GTPases such as Rac, p21-activated kinase (PAK) family members and neuronal Wiskott-Aldrich syndrome protein (N-WASP) [6], all of which are recruited into a complex with DCC in commissural neurons following stimulation with netrin-1 [7]. Although these and other downstream components of netrin-1 and DCC signaling have been identified [8], the subcellular distribution of this activation in neurons remains poorly understood. Here, using a genetically encoded biosensor and fluorescence-lifetime imaging microscopy-Förster resonance energy transfer (FLIM-FRET) imaging [9,10], we visualize rapid netrin-1 induced activation of Cdc42 localized to the growth cones of embryonic rat spinal commissural neurons. We then investigated the intracellular signaling mechanism involved, demonstrating that netrin-1 rapidly recruits DCC to the leading edge of commissural neuron growth cones and that src family tyrosine kinase (SFK) activation is required for netrin-1 activation of Cdc42 in neuronal growth cones.

## Materials and Methods

### Microdissection and culture of embryonic rat spinal commissural neurons

All procedures were performed in accordance with the Canadian Council on Animal Care guidelines for the use of animals in research and were approved by the Montreal Neurological Institute Animal Care Committee. Staged pregnant Sprague-Dawley rats were obtained from Charles River Laboratories Canada (St Constant, QC) and killed by carbon dioxide overdose followed by cervical dislocation. Embryos were obtained by Caesarian section and embryonic day 13 (E13; vaginal plug is E0) dorsal spinal cords isolated and dissociated as described [11]. Neurons were plated at 350,000 cells/well in 35 mm, 14 mm glass No. 1.5 thickness Glass Bottom dishes (MatTek Corporation, MA, USA) coated with 2 μg/mL poly-D-lysine (PDL) in medium composed of Neurobasal, 10% fetal bovine serum (FBS), 1% GlutaMAX (Invitrogen) and 1% penicillin/streptomycin (Invitrogen). Following 1 day *in vitro* (DIV), medium was replaced by Neurobasal, 2% B27 (Invitrogen), and 1% GlutaMAX (without antibiotics). After 4–6 hours, cells were transfected using 1.9 μL of Lipofectamine 2000 (Life Technologies) and 2.5 μg DNA per well, according to the manufacturer's protocol. Culture medium was not changed following transfection. On subsequent days, 200 ng/mL purified recombinant netrin-1 [12] was applied for 5 min before fixation with 4% PFA at 37°C for 30 min and then washed with PBS. For SFK inhibition assays, 10 μM PP2 or PP3 (Calbiochem) was added to the cells 15 min before netrin-1 stimulation. To immunocytochemically label plasma membrane DCC, 5 min following the application of 200 ng/mL netrin-1 cultures were fixed with ice cold 4% PFA in PBS in the absence of detergent. DCC was labeled with a monoclonal antibody specific for an extracellular epitope (monoclonal AF5, Calbiochem USA). Growth cone peripheral (P) and central (C) domains were marked by an observer blinded to the experimental conditions. Images were collected using an Axiovert 100 epifluorescence microscope (Zeiss, Oberkochen, Germany) with a Magnafire CCD camera (Optronics, Goleta, CA)

### Confocal microscopy imaging

Fluorescence microscopy images were acquired with a Zeiss 710 confocal microscope equipped with a C-Apochromat 40x/1.2 N.A. W Korr M27 objective, with the following settings: image size 512x512 pixels, 12 bits, pixel dwell time 6.3 μs, zoom 2, pixel dimension 208 nm, laser CW 440 nm at 1%, PMT 600 V, gain 1, offset 20, dichroic mirror MBS-445 and bandpass emission filter 454–560 nm.

### FRET reporter probe

**Fig 1:**
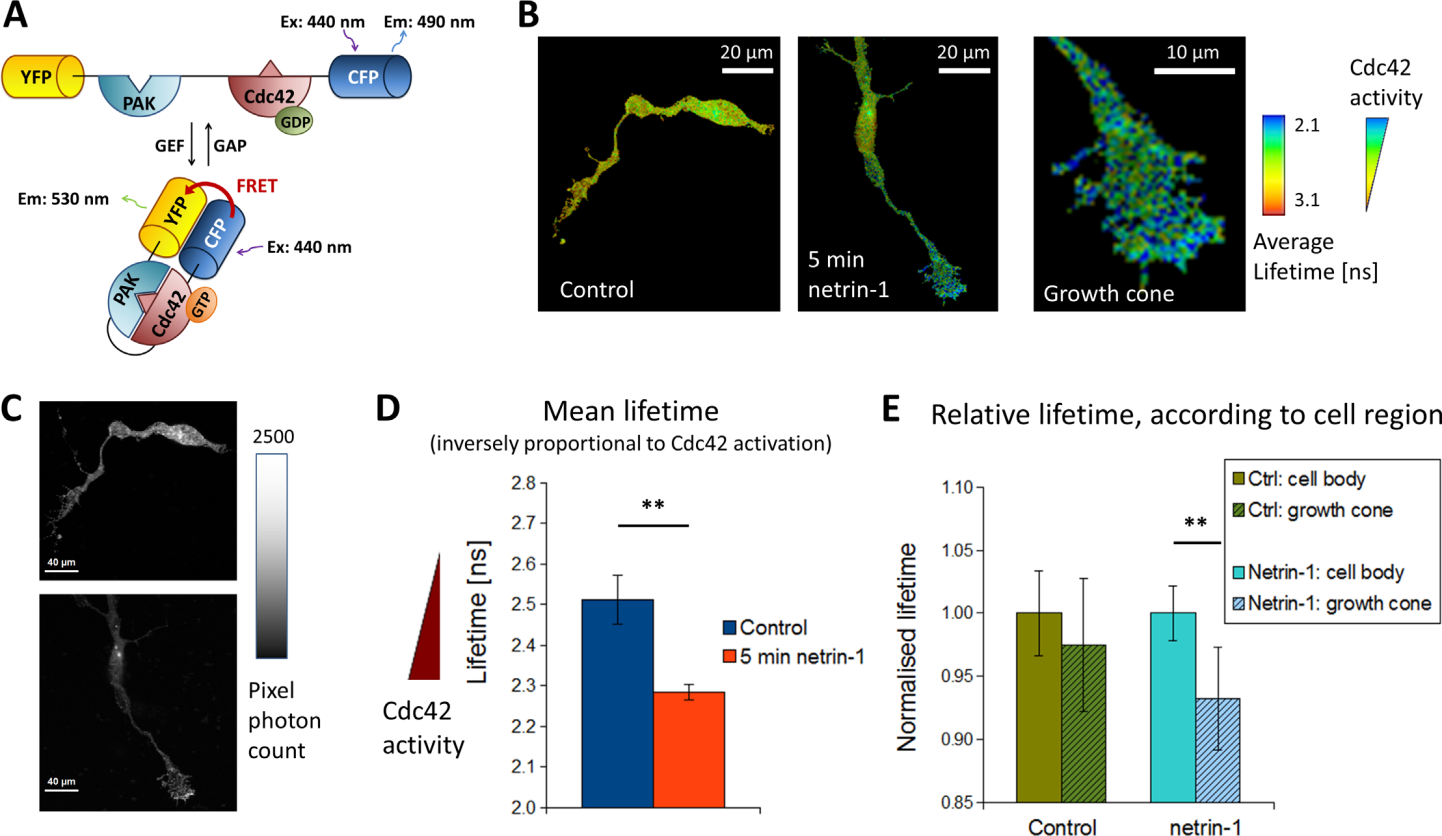
Netrin-1 activates Cdc42 in neuronal growth cones. A. Schematic representation of the Cdc42 Raichu FRET construct. Interacting domains of Cdc42 and its binding partner PAK1 are flanked by modified CFP and YFP fluorophores (SECFP and Venus, respectively). B. Heat maps of the lifetime distribution in cultured embryonic spinal commissural neurons in control (left) and netrin-1 (right) stimulated conditions. As indicated by the color bar on the right, average lifetime is inversely proportional to the extent of Cdc42 activation. C. Photon count (per pixel) of the two neurons in B. showing the distribution of the FRET reporter throughout the cell. D. Bar graph of the mean lifetime in the control and netrin-1 condition. A significant decrease in lifetime (corresponding to an increase of Cdc42 activation) was detected within 5 min after stimulation by 200 ng/mL netrin-1 (p = 0.0012), ctrl, n = 19; netrin-1, n = 26. Error bars are SEM. E. Parsing the level of activation in the cell body compared to the growth cone, cell by cell, no significant difference was detected in control conditions. A significant decrease in lifetime, corresponding to increased Cdc42 activation, was detected when comparing the growth cone to the cell body in the netrin-1 stimulated condition (p = 0.009, ** indicates p < 0.01, Student's paired t-test; error bars are SEM, ctrl, n = 6; netrin-1, n = 10; ns: nanoseconds).

The Cdc42 Raichu FRET reporter construct was provided by Dr. M. Matsuda (Kyoto University). It is composed of four domains: the interacting domains of Cdc42 and its binding partner PAK1 which are flanked by modified CFP and YFP fluorophores (SECFP and Venus, respectively; see Fig 1A). In the un-activated state the SECFP and Venus domains are sufficiently distant that they do not interact, but when the probe is activated, for instance when recruited to DCC, the configuration changes and SECFP and Venus are brought sufficiently close to allow FRET to occur. FRET describes dipole-dipole energy transfer between a donor and acceptor dye pair that only occurs when they are in close proximity (typically less than 5–10 nm depending on the Förster radius for the pair). Typically FRET is calculated by measuring the fluorescence intensity ratio of the acceptor (Venus) to the donor (SECFP) chromophore. FLIM-FRET instead measures the lifetime of the donor (SECFP) molecule, which decreases when FRET occurs. This is spatially mapped via time resolved imaging to resolve the area of activity of the FRET reporter probe.

### FLIM settings

Fluorescence lifetime images were measured with a PicoQuant (Germany) time-correlated single photon counting (TCSPC) device coupled to the Zeiss 710 confocal microscope. Images were recorded using the following settings: image size 256x256 pixels, zoom 2, pixel dimension 416 nm, pixel dwell time 6 μs, 440 nm laser in pulse mode at 50 MHz with 33% power. Photons were acquired for 30–60 s using the provided PicoQuant CFP emission filter. This duration enabled the collection of more than 1000 photons per pixel, thereby allowing obtaining relevant measurement in all cell regions. Data acquisition for TCSPC FLIM is slow (minutes time scale), which makes it difficult to measure fast dynamics in living samples at physiological temperatures. Therefore all experiments were conducted on fixed cells. Fixation has been shown to not affect the lifetime measurement [13]. This also allows greater precision regarding the length of treatment.

### IRF calibration

To obtain more accurate lifetime results, the instrument response function (IRF) was measured prior to experimental imaging. This improves the accuracy of the results and fits as compared to using an estimated IRF. Various options exist to measure the IRF [14,15]. We applied a protocol using KI quenched fluorescein [15] that has the advantage of measuring IRF in the exactly same configuration as that used to image the sample (fluorescein has spectral properties similar to CFP). The fluorescence lifetime was then measured using the same settings as for the experimental condition.

### Lifetime calculation

Lifetime images show “fast-FLIM” which is the mean photon arrival time for each pixel and were used as a preview. For the actual FLIM calculation, the photon arrival times were pooled for the regions of interest (the whole cell, the cell body, or the growth cone) and the fluorescence lifetime decays were fitted using two exponential components and the measured IRF. Two components were required, but sufficient, to obtain good fits (low χ^2^). The mean fluorescence lifetime was calculated according to:

$${<\tau }_{f}>=\frac{\sum_{n=1}^{2}A_{n}*{\tau_{n}}^{2}}{\sum_{n=1}^{2}A_{n}*\tau_{n}},$$

where *A*_*n*_ and *τ*_*n*_ are the amplitude and lifetime of the n^th^ component. <τ_f_> is the average time during which the emitters (e.g. fluorophores) remain in their excited state after the onset of excitation.

## Results

### Netrin-1 activates Cdc42 in commissural neuron growth cones

Netrin-1 is an essential extracellular chemoattractant that guides axonal extension by DCC expressing commissural neurons in the embryonic spinal cord [5,8]. To address the temporal and spatial activation of signal transduction mechanisms activated by netrin-1 downstream of DCC, we utilized a CFP/YFP FRET biosensor for the Rho GTPase Cdc42 [16,17]. The cDNA encoding the reporter was transfected into E13 rat spinal commissural neurons (1 DIV) isolated and cultured as described [7,11]. When grown in dispersed cell culture, as described here, embryonic rat spinal commissural neurons respond to netrin-1 as a chemoattractant for up to at least 4 DIV [18], and rapidly respond to bath application of netrin-1 with increased numbers of filopodia and growth cone expansion, characteristic of membrane extension evoked by a chemoattractant [7]. At 2 DIV, we imaged biosensor activation using a Zeiss LSM 710 confocal microscope and a variation of standard FRET, FLIM-FRET [14]. FLIM-FRET maps the distribution of fluorescence lifetimes across an image and is sensitive to small changes in this parameter. In contrast to classical intensity ratiometric-FRET, FLIM-FRET, is independent of the concentration of the fluorophores and is not affected by photobleaching. Nevertheless we always acquired at least 1000 photons per pixel to rule-out any variability in the measurement that could have been produced by a low level of reporter in subregions of a cell. Fig 1C illustrates that photons were readily detected from all regions of the cell indicating distribution of the probe throughout the cell. Additionally, only the lifetime of the donor fluorophore is measured when using FLIM-FRET, allowing relatively straight-forward and rapid recording.

Bath application of 200 ng/mL netrin-1 to commissural neurons revealed rapid activation of the Cdc42 biosensor in the growth cones (Fig 1). Importantly, this finding is consistent with previous biochemical assays of netrin-1 induced activation of Cdc42 in commissural neurons [7]. In spite of the bath application of netrin-1, our findings reveal subcellular specificity of Cdc42 activation limited largely to the axonal growth cone.

### Netrin-1 enriches DCC in the growth cone peripheral domain

**Fig 2:**
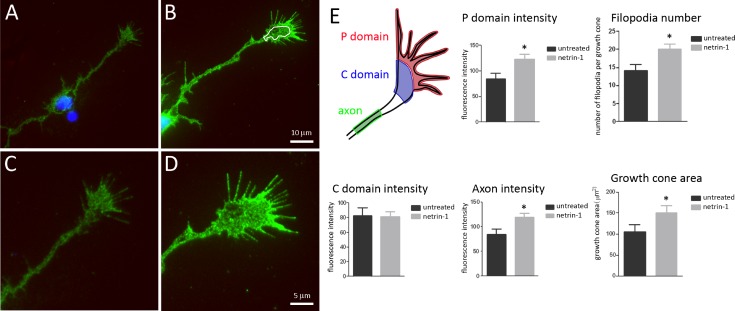
Netrin-1 rapidly enriches DCC in the growth cone peripheral domain. Cultured embryonic rat spinal commissural neurons at 2 DIV were treated with 200 ng/mL netrin-1 for 5 min, fixed without permeabilization, and immunostained to label plasma membrane DCC. Panels A and B show DCC immunoreactivity (green) at the surface of the cell body, axon and growth cone of a control untreated neuron (A) and of a neuron treated with netrin-1 (B). Cells were imaged while maintaining the same exposure for all micrographs. Panels C and D illustrate magnified images of the growth cones in panels A and B respectively. The growth cone schematic in E illustrates the regions in which DCC florescence was quantified: Peripheral (P) domain, Central (C) domain, and along the axon. White lines in panel B delineate the boundaries between the P and C domains of the growth cone. The bar graphs show the average florescence intensity in arbitrary units of these regions, filopodia number, and surface area of treated and untreated growth cones. Hoechst stain was applied to label nuclei blue. Scale bar corresponds to 10 μM in panels A and B, and 5 μM in panels C and D. (* indicates p < 0.05, Student’s t-test; error bars are SEM; untreated, n = 19; netrin-1, n = 16).

We then examined the distribution of DCC in these neurons following stimulation with netrin-1. Five min following bath application of 200 ng/mL netrin-1, cultured embryonic spinal commissural neurons were fixed without permeabilization. Cells were then labeled immunocytochemically using a monoclonal antibody against an extracellular epitope of DCC, again in the absence of detergent to prevent membrane permeabilization. Relative levels of DCC immunofluorescence were visualized by imaging all growth cones using the same microscope, light intensity, and camera exposure (Fig 2). DCC immunoreactivity was associated with the cell body, along the axon, and within the growth cone of commissural neurons. Application of netrin-1 did not measurably change the average intensity of DCC immunoreactivity detected within the central (C) domain of the growth cone (Fig 2). In contrast, in cells treated with netrin-1, DCC was significantly enriched in the growth cone peripheral (P) domain, indicating a rapid recruitment of DCC to the leading edge by netrin-1. In some cases Cdc42 activation appeared maximal at the leading edge of the growth cone (Fig 1B); however, this was not consistent in all growth cones examined.

We have previously documented that netrin-1, either bath applied or presented as a substrate, rapidly evokes an increase in the number of filopodia per growth cone, and in growth cone surface area, termed growth cone expansion [7]. Consistent with this, we detected increases in both filopodia number and growth cone surface area within 5 min following bath application of netrin-1. Netrin-1 treatment resulted in an increase in plasma membrane DCC along the length of the axon to an extent similar to that detected in the growth cone. Although netrin-1 has been shown to promote axon branch formation by embryonic cortical neurons [19,20], commissural neurons typically extend a long unbranched axon in the embryonic spinal cord and netrin-1 induction of commissural axon branching has not been reported. Consistent with this, and with our focus on early events triggered by netrin-1 application, we did not detect new branches formed by the spinal commissural axons.

### Netrin-1 activation of Cdc42 requires SFK signaling

**Fig 3:**
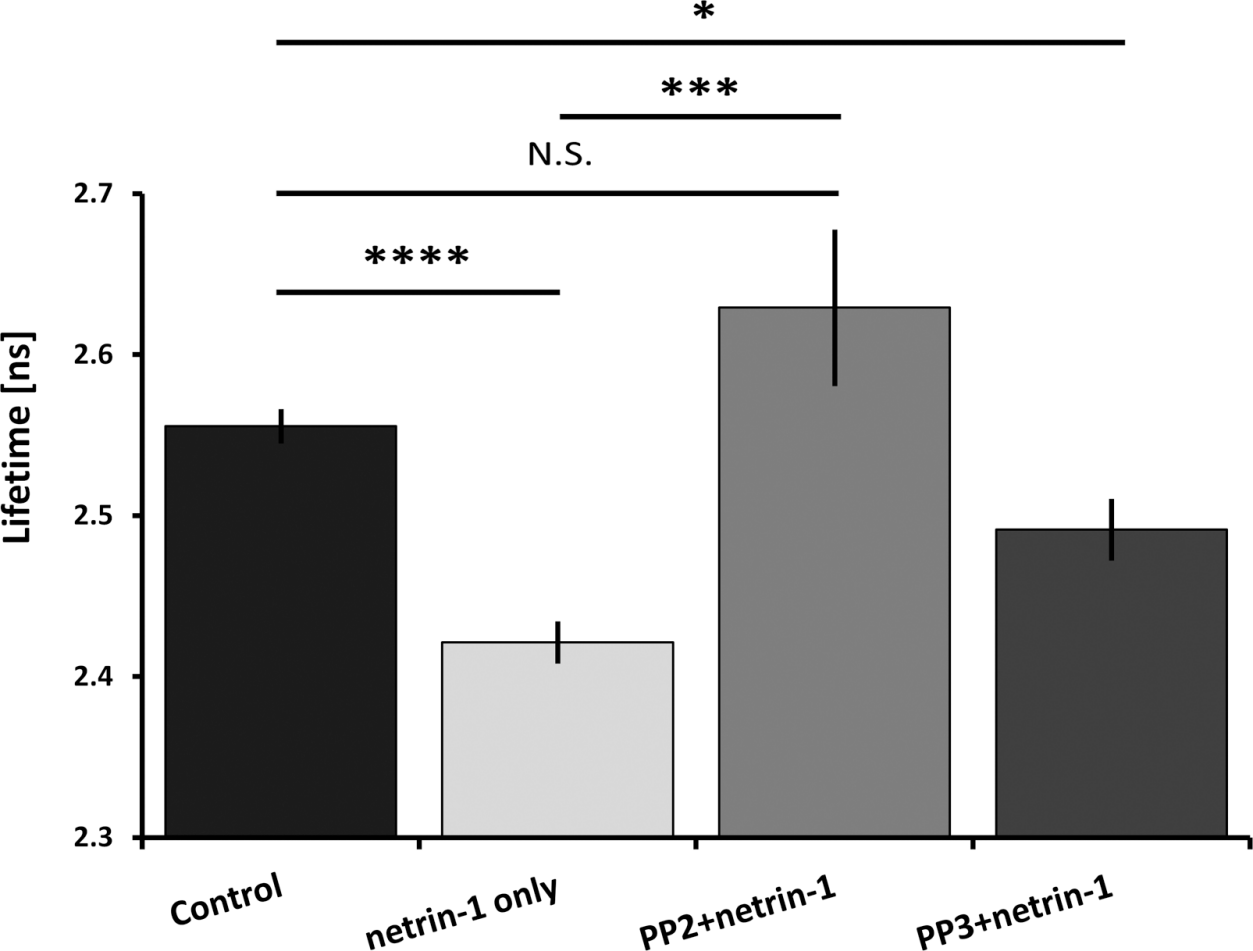
Inhibition of SFK signaling blocks netrin-1 activation of Cdc42 in neuronal growth cones. The src-family tyrosine kinase inhibitor PP2 blocks Cdc42 activation as measured using FLIM-FRET in commissural neurons. The bar graph illustrates the mean Cdc42-fluorescence lifetime measured in different treatment conditions. The conditions are: Control with no stimulation; netrin-1 only, 5 min of 200 ng/mL netrin-1; PP2, SFK inhibitor. PP3: negative control and inactive analogue of PP2. PP2 and PP3 were added at 10 μM for 15 min before bath application of 200 ng/mL netrin-1. PP2 blocked the increase in Cdc42 activation in growth cones while application of PP3 had no effect. (* indicates p < 0.05, ** p<0.01, *** p < 0.001, **** p < 0.0001, N.S.: not significant, unpaired t-test with two-tailed distribution computed using Excel TTEST function TTEST (array1,array2,2,2); error bars are SEM, control untreated, n = 9;; netrin-1, n = 10; PP2, n = 8; PP3, n = 10; ns: nanoseconds).

Netrin-1 binding to DCC activates SFK signaling in neurons [21–24]. SFKs regulate Rho family GTPase activation [25] and are therefore candidates to activate Cdc42 in response to netrin-1. We therefore employed the cell permeable SFK inhibitor PP2, and its inactive analogue PP3 as a control [26], to test the biochemical specificity of the netrin-1 induced increase in Cdc42 activation. Inhibition using PP2, applied 15 min before bath application of 200 ng/mL netrin-1, blocked the increase in Cdc42 activation in growth cones detected using FLIM-FRET, while application of PP3 did not (Fig 3). Our findings support the conclusion that netrin-1 signaling via DCC activates a SFK that is essential for the downstream activation of Cdc42 in embryonic spinal commissural neuron growth cones.

## Discussion

Lamellipodia and filopodia form the dynamic F-actin based leading edge of an axonal growth cone [27,28] and are required for axon guidance [29–32]. Notably, contact of the tip of a single filopodium with an appropriate target can reorient axon growth [31,33], consistent with the enrichment of receptors for guidance cues, such as DCC, at the tips of growth cone filopodia [7]. The cytoskeletal changes underlying axon outgrowth and the morphology of axonal growth cones are regulated by the Rho family of small GTPases [34].

Among the Rho GTPases, Cdc42 is a critical regulatory nexus for cell polarity [6]. Although Cdc42 is dispensable for movement in many cell types, it is essential for directional movement, providing a functional dissociation between the ability to move and the capacity to move in a particular direction [6,35]. Subcellular accumulation and activation of Cdc42 is a mechanism of polarization and directional migration that is conserved from budding yeast to migrating lymphocytes. Initial functional studies, carried out primarily in fibroblast cell lines, demonstrated that Cdc42 promotes filopodia formation [36] and the extension of a leading edge by activating Rac and WASP, resulting in Arp2/3 activation and the nucleation of actin filaments [37,38]. Subsequent imaging studies revealed that Cdc42 and Rac1 activation gradually increase toward the leading edge of motile cells, with Cdc42 activation prominent at the tip of the leading edge [17], consistent with its role directing motility.

The netrin-1 receptor DCC is highly enriched in axonal growth cone filopodia and exerts a potent influence on the reorganization of F-actin in response to netrin-1 [7,12,39]. In initial biochemical studies using cell lines, netrin-1 signaling through DCC was found to activate Cdc42 and Rac1 to trigger filopodia formation and cell spreading [12,40]. It was subsequently determined that bath application of netrin-1 to embryonic rat spinal commissural neurons triggers growth cone expansion, a rapid almost two-fold increase in growth cone surface area and number of filopodia that is dependent on the activation of Cdc42 and Rac1 [7]. In biochemical studies of commissural neurons, netrin-1 signaling through DCC was shown to recruit and activate Cdc42, Rac1, and PAK1, and provided evidence that Cdc42 activation is upstream of Rac1 in these cells [7]. To assess the functional significance of these proteins, the expression of dominant negative Cdc42, Rac1, or N-WASP was demonstrated to significantly reduce netrin-1 induced growth cone expansion [7].

Although Rac function has been investigated downstream of netrin-1 and DCC [7,12,41–49]; study of Cdc42 function in this context has been relatively limited [7,12,46], in spite of its critical role directing motility. Here, using FLIM-FRET, we detected rapid netrin-1 induced activation of Cdc42 in commissural neuron growth cones, but not in the cell body, demonstrating subcellular specificity. While some growth cones exhibited clear enrichment of activated Cdc42 associated with the leading edge (Fig 1B), this was not sufficiently consistent to reveal statistically significant spatial specificity within growth cones. To further address the temporal and spatial activation of Cdc42 by DCC within neuronal growth cones, it will be critical in future studies to examine a time course of Cdc42 activation following netrin-1 stimulation that is restricted to a sub-portion of the growth cone.

Despite the non-directional bath application of netrin-1, we detected a rapid, netrin-1 induced recruitment of DCC to the plasma membrane of the growth cone P domain, coincident with increased numbers of filopodia and larger growth cone surface area. Netrin-1 added in solution is rapidly adsorbed to a poly-lysine coated cell culture substrate [7,39,50] and immobilization to a substrate is strongly implicated in the capacity of netrin-1 to direct growth cone motility [7,39,50–52]. We speculate that as the growth advances DCC is selectively enriched at the leading edge by binding substrate bound netrin-1. Although the underlying mechanism remains to be addressed, the local enrichment of DCC could involve lateral redistribution in the plasma membrane, or recruitment from intracellular cargo vesicles containing DCC [39,53–57]. Consistent with our current findings, previous studies in *C*. *elegans* and *Drosophila* have provided evidence that the extracellular distribution of netrin can orient the subcellular distribution of the DCC orthologs UNC-40 and Frazzled [58–60]. Evidence has also been provided that plasma membrane recruitment of DCC by netrin-1 in mammalian neurons may contribute to neocortial axon branching [57]. Application of netrin-1 increases exocytosis in neuronal growth cones through a mechanism that requires SNARE function and activation of Erk1/2 and SFKs [61,62]. Additionally, activation of PKA recruits DCC from intracellular vesicles to the neuronal plasma membrane, enhancing axon extension and chemotropic turning to netrin-1 via a mechanism dependent on the v-SNARE VAMP2 [53,63]. DCC also forms a complex with the t-SNARE synatxin-1 and v-SNARE TI-VAMP, and trafficking via these interaction has been implicated in netrin-1 mediated guidance [64,65]. Application of netrin-1 alone increases the amount of DCC at the plasma membrane and recruitment of DCC containing vesicles may contribute to this increase [53,55,57,65]. Interestingly, Cdc42 regulates the polarized recruitment of vesicles to the neuronal plasma membrane [66], suggesting that netrin-1 mediated activation of Cdc42 by DCC might promote the local recruitment of DCC, but the possible contribution of this mechanism to DCC trafficking has not been addressed.

Temporally and spatially restricted activation of Cdc42 is both necessary and sufficient to trigger cell polarization in many cell types [6]. Spatially restricted local activation of Cdc42 is typically controlled by the local recruitment and enrichment of both Cdc42 itself and a guanine nucleotide exchange factor (GEF), which functions as an upstream activator for Cdc42 [25]. Our current results, and previous findings [7], provide strong evidence that netrin-1 activates a GEF for Cdc42. Netrin-1 activation of Cdc42 in neurons was demonstrated using GTPγS, which cannot be converted to GDP and binds irreversibly to the Rho-GTPases. Commissural neuron lysates were incubated with GTPγS, followed by the isolation of endogenous Cdc42 bound to GTPγS to reveal increased activation by netrin-1 [7]. Importantly, because this assay measures the accumulation of non-hydrolysable GTPγS bound to Cdc42, the increased binding strongly implicates netrin-1 in the activation of a GEF for Cdc42 in primary commissural neurons.

The SFK fyn is activated by netrin-1 downstream of DCC and focal adhesion kinase (FAK) [21–24]. SFKs regulate several GEFs that function as upstream activators of Rho GTPases [25] and therefore SFK activation downstream of DCC is a candidate activator of Cdc42 in response to netrin-1. Although a GEF for Cdc42 that is activated by netrin-1 has not yet been reported, SFK activation of a GEF is consistent with our demonstration that Cdc42 activation is blocked by the SFK inhibitor PP2.

Cdc42 function in neural cells is not limited to axonal growth cones. In hippocampal neurons, Cdc42 activation has been imaged in individual activated dendritic spines, suggesting roles for Cdc42 activation in spine motility and synaptic plasticity [67]. Furthermore, disruption of Cdc42 function in the nervous system has been implicated in Alzheimer’s disease [68], schizophrenia [69], epilepsy [70,71] and neurofibromatoses [72]. Netrin signaling is similarly implicated in synaptogenesis [55], synaptic plasticity [73], Alzheimer's disease [74–76], schizophrenia [77], and brain tumor cell biology [78–81], suggesting that the significance of netrin-1 signaling via Cdc42 likely extends beyond roles directing chemotropic axon guidance.
